# Dockomatic - automated ligand creation and docking

**DOI:** 10.1186/1756-0500-3-289

**Published:** 2010-11-08

**Authors:** Casey W Bullock, Reed B Jacob, Owen M McDougal, Greg Hampikian, Tim Andersen

**Affiliations:** 1 Computer Science Department, Boise State University, Boise, Idaho 83725, USA; 2 Department of Chemistry and Biochemistry, Boise State University, Boise, Idaho 83725, USA; 3 Department of Biological Sciences, Boise State University, Boise, Idaho 83725, USA

## Abstract

**Background:**

The application of computational modeling to rationally design drugs and characterize macro biomolecular receptors has proven increasingly useful due to the accessibility of computing clusters and clouds. AutoDock is a well-known and powerful software program used to model ligand to receptor binding interactions. In its current version, AutoDock requires significant amounts of user time to setup and run jobs, and collect results. This paper presents DockoMatic, a user friendly Graphical User Interface (GUI) application that eases and automates the creation and management of AutoDock jobs for high throughput screening of ligand to receptor interactions.

**Results:**

DockoMatic allows the user to invoke and manage AutoDock jobs on a single computer or cluster, including jobs for evaluating secondary ligand interactions. It also automates the process of collecting, summarizing, and viewing results. In addition, DockoMatic automates creation of peptide ligand .pdb files from strings of single-letter amino acid abbreviations.

**Conclusions:**

DockoMatic significantly reduces the complexity of managing multiple AutoDock jobs by facilitating ligand and AutoDock job creation and management.

## Background

Several computational modeling programs have been developed to estimate ligand binding efficacy by modeling atomic interactions between the ligand and a target receptor [1,2], including AutoDock [1,3], MOE-Dock [4], GOLD [5], DOCK [6], and Glide [7]. Of these, AutoDock is one of the most widely used tools for simulating the docking of ligands to receptors [1]. AutoDock, and other similar programs, rank ligands by estimating the ligand to receptor binding interaction energy [8]. The strength of AutoDock is the computational algorithm, which uses a combination of linear regression analysis and the AMBER force field. The AutoDock application works very well for the analysis of a single ligand with a specified receptor. However, for multiple screening of peptide ligands binding to a protein receptor, it is necessary to run ligands individually through AutoDock, followed by manual analysis of the output file to confirm ligand interaction results. This process is time consuming in both computation and required user involvement. This paper presents DockoMatic, a GUI application designed to facilitate the use of AutoDock by automating the setup, submission, and management of AutoDock jobs, and summarizing and easing analysis of results.

DockoMatic was conceived as a joint project with the Departments of Computer Science and Chemistry and Biochemistry, and Biologal Sciences at Boise State University, as a way to simplify and speed the process of creating peptide ligands and simulating the docking of those ligands to receptors. DockoMatic's intuitive user interface greatly reduces the amount of user time required to setup, submit, and analyze AutoDock jobs.

DockoMatic has the following major features:

• Intuitive GUI for user controlled automation

• Create, submit, and manage AutoDock jobs

• Setup and management of competitive binding experiments

• Automatic generation of bound receptor structure for competitive binding experiments

• Peptide-based ligand creation based on single letter residue codes

• Job tracking

• Easily viewable result report

• Result summarization, screening, and analysis

While other tools are available for using AutoDock on clusters of computers [9], no tool that we are aware of includes all of the features of DockoMatic in a single package, and no tool has automated the time consuming creation of peptide-based ligand files.

## Implementation

### Intuitive GUI for user controlled automation

DockoMatic was designed to have a relatively simple and intuitive interface for use by the chemist or biochemist that has limited computer science training. It does not require involved scripting, or command line processing. Instead, the user interface has been designed to lead the user through the requirements for a successful AutoDock job creation, submission, and analysis. DockoMatic's design layout places the user required items on the left, the job information or management grid in the center, and the program options on the right. The left side of the window detailing user input requirements begins with the output directory. The user clicks on this box and navigates to the directory where the user wants DockoMatic to place the results. If no output directory is specified, the output directory defaults to the directory the user is in when the GUI is started. The ligand box is where the user can either select a single .pdb file, or input a string of amino acids. For high-throughput screening, instead of entering an individual ligand the user may enter a file name and check the box for "Use Ligand List File". In this case, the file name must refer to an input file that contains either a list of amino acid strings, or paths to existing ligand .pdb files. In a similar manner, the user selects both the Receptor and box coordinate files. Users may also choose to specify a secondary ligand or a file containing a list of secondary ligands to model how an additional ligand may bond in the presence of the first ligand.

After entering these items, the user can create AutoDock jobs by pressing the "New Job" button. This populates the management grid with a list of all jobs. The total number of jobs created is equal to the cross product of the ligands and box coordinates. At this point the job specifications can be manipulated before the jobs are started. If the job details are satisfactory, the "Start All Jobs" button can be selected and all jobs will be started. If the user wishes to start an individual job, the user does so by selecting the desired job and pressing the "Start Selected" button. Jobs may be stopped and removed from the management grid with either the "Remove All Jobs" or "Remove Selected" buttons.

The management grid lists the job number, ligand specified or path to .pdb file, output directory path, path to receptor, path to box coordinate file, secondary ligand or path to secondary ligand file, whether the job is a swarm job, and the current status of the job. Swarm can be specified, via a check-box, for parallel job submission to a cluster, or jobs can be spawned as individual processes on a single workstation. Once jobs are started, the status of each job in the window is automatically checked every ten seconds. Below the management grid is the "messages box" relating DockoMatic's progress information.

As DockoMatic detects that jobs are complete, the ability to view the job result using PyMOL is enabled (requires that PyMOL be installed) [10]. Two buttons provide the PyMOL functionality. The "View file with PyMOL" button can be used to view a single .pdb file. If an entry in the management grid is highlighted, the file chooser box will open to the output directory specified for the job. The user can then browse to the appropriate directory and choose a .pdb file to view. The "View All PDB Files" button has similar behavior. If a job is highlighted when this button is pressed, all .pdb files in all sub-directories of the output directory are loaded into PyMOL. If no job entry is highlighted, the user is able to browse to the output directory desired. After the directory is selected, the behavior is the same as the other PyMOL button.

**Figure 1:**
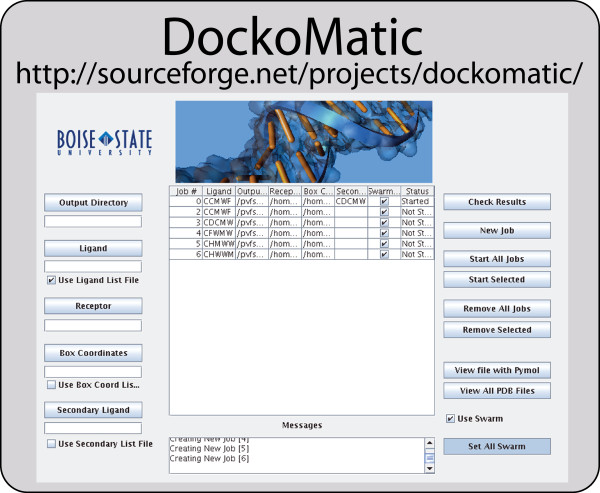
**DockoMatic GUI interface**. The Graphical User Interface for DockoMatic with user input fields (left), current processing status (center), and results/analysis fields (right).

See Figure 1 for details.

### Create and submit AutoDock jobs

While DockoMatic significantly reduces the time required by the user to create and submit jobs to AutoDock, there are a few files the user must provide. These include:

1) the ligand .pdb file or sequence

2) a receptor .pdb file

3) a user defined template .gpf file

The template .gpf input file required by DockoMatic only needs to have defined the specific grid box coordinates for the region of interest. DockoMatic then automates creation of the fully prepared .gpf file that is required by AutoDock, which includes the following information:

• The specific atom types to be calculated as maps by AutoGrid, taken from the already prepared ligand .pdbqt file.

• The location of the prepared receptor .pdbqt file.

• Specific grid box coordinates.

Automating the creation of the fully prepared .gpf saves the user time, as this is typically done manually using AutoDock Tools. User specified Cartesian map coordinates presented in the template .gpf file are required to determine the area of interest on the receptor. However, DockoMatic hides the preparation of the ligand and receptor, as well as the creation of the prepared .gpf file, from the user.

AutoDock job submission and processing of ligand and receptor .pdb files results in their conversion to the .pdbqt file format. It is also necessary to prepare the ligand specific box coordinate file as well as create the "docking parameter file," for AutoDock. DockoMatic automates the preparation of these files via MGLTools [11].

By itself AutoDock does not possess the functionality to efficiently setup and process the binding of multiple ligands to a receptor simultaneously, nor can it directly accommodate combinations of different ligands, receptors, and grid box locations. In order to ease setup of multiple jobs, DockoMatic processes lists of ligands and box coordinates for the desired receptor, followed by automatic job creation for each possible combination of ligand, receptor, and grid box coordinate. For example, supplying a list of 10 peptide ligands, one receptor, and three different box coordinate files results in (10 × 1 × 3) = 30 different jobs being created. Through the DockoMatic interface the user can then edit this job list, select jobs, and queue them for batch processing.

AutoDock runs 10 stochastic simulations per compound to find the best docking site, as its default setting. Running 10 simulations occurs quickly, but it has the disadvantage of returning less accurate results than longer runs of 50 to 100 simulations. AutoDock documentation recommends using at least 50 docking simulations to ensure accurate results with the added comment that more dockings are more likely to result in better statistical results. By default, DockoMatic performs 100 simulations, a number consistent with that reported by others in the literature, to provide a good compromise between speed and accuracy [12].

### Multiple ligands for competing binding sites

A common approach to competitive binding of multiple ligands (A and B) using AutoDock involves creating a new protein complex receptor from the results of an AutoDock job with ligand A and the original receptor. The binding interaction coordinates for ligand A are generally determined by the conformation with the lowest binding energy. The resulting receptor is then subjected to a binding run with ligand B in the traditional manner. The result provides a determination of binding energy for ligand B with ligand A already bound. Unfortunately, AutoDock does not automate the binding of a ligand conformation to a receptor, nor automatically start a second job, which means this type of experiment is time consuming and laborious.

DockoMatic automates competitive binding experiments, with ligand B supplied in the "Secondary Ligand" box of the GUI. The process proceeds as follows. Ligand A from the "Ligand" box in the GUI is created or used, and an AutoDock job is run as normal. After this job is finished, DockoMatic creates a new combined protein complex from the highest ranking conformation of ligand A and the original receptor. Next, a second AutoDock job is automatically started by DockoMatic with the "Secondary Ligand" and the new receptor.

### Peptide-based ligand creation

AutoDock requires all ligand coordinate files to be submitted in .pdb format. This is not a problem if the .pdb files already exist, but if they do not exist then the creation of novel ligand structures can be tedious. DockoMatic automates peptide-based ligand creation either as a prelude to creating an AutoDock job, or as its primary function. DockoMatic constructs a .pdb file for a ligand based on the user supplied string of alphabet characters representing the single letter amino acid sequence of the ligand (e.g. WCWKW). This is a time saving measure that facilitates job setup. DockoMatic creates peptide ligands using pre-created .pdb files. The algorithm for creating a ligand structure from a peptide ligand string can be summarized as follows.

1. add beginning

2. if next amino acid is not proline, add backbone structure

3. add amino acid sidechain

4. repeat steps 2 and 3 until the ligand string is exhausted

5. add end

6. optimize ligand structure

The beginning step starts the chain of amino acids with a hydrogen atom, while the end step terminates the chain with a hydrogen and an oxygen atom. Proline was treated separately from the other amino acids due to the bend that may be associated with the presence of this amino acid in the backbone of a peptide or protein. When proline is encountered, we do not add a backbone structure since the backbone is already built into the side chain .pdb file. To avoid atoms being set too closely, the orientation of each sidechain-backbone pair alternate up and down. In total, there are 44 .pdb files used for ligand creation; one each for the beginning and end, a backbone in two orientations, and forty side chains to represent each of the twenty amino acids in the up and down position. As a last step, the ligand structure that DockoMatic creates is optimized using Obconformer, a tool supplied in the OpenBabel package [13].

### Job tracking in parallel on a cluster

Another feature of DockoMatic is the ability to create swarm jobs to facilitate running of multiple docking jobs in parallel on a cluster [14]. While it is not a requirement to run DockoMatic on a cluster, the use of one greatly decreases the amount of time required to spawn and complete multiple jobs. This was the type of usage in mind when the application was designed. The speedup when processing jobs in parallel on a cluster scales linearly with the number of machines, since jobs can run independently.

### Easily viewable results

DockoMatic parses, summarizes, and simplifies AutoDock results for the user. The results of AutoDock are output in the form of a single .dlg file, with the size of the file dependent upon the number of simulations specified by the user. The .dlg file is only accessible to the user through AutoDock Tools, complicating the abstraction and viewing of promising individual results. Summary output from DockoMatic includes separate ligand .pdb files for each simulation in addition to a summary of the binding energy, inhibition constant, conformation statistics, and cluster rank. DockoMatic correlates the result information for each simulation into a single file that serves as the source file for data ranking. The .pdb file with the highest rank (1 being the highest) represents the ligand to receptor combination with the lowest binding energy and is generally considered to be the most favorable binding model.

### Result screening and analysis

To further reduce the time required for data analysis, DockoMatic provides a results check button, which takes advantage of a second user supplied .gpf file. The second .gpf file can be used to screen results when a ligand binding domain is assumed based on experimental evidence. The smaller grid encompasses the active site amino acids known to be significant for ligand binding. From this second box, DockoMatic screens the results and outputs the best and average values of both estimated binding energy and the estimated inhibition constant. The output from this process includes: 1) the percent of runs that lie inside the secondary gpf coordinates, 2) the average and best binding energy, and 3) the average and best inhibition constant. This information is formatted in a simple text file similar to the ranked results list mentioned above. This feature is of particular use for study of the entire receptor surface to determine the likelihood for a ligand to bind to a particular binding site, and reduces the user time required to visually analyze all output files.

## Results and Discussion

To assess the performance of DockoMatic, the following major functionality was evaluated on a specific set of docking problems: 1) automated peptide-ligand creation; 2) concurrent job submission; 3) user required processing time.

**Figure 2:**
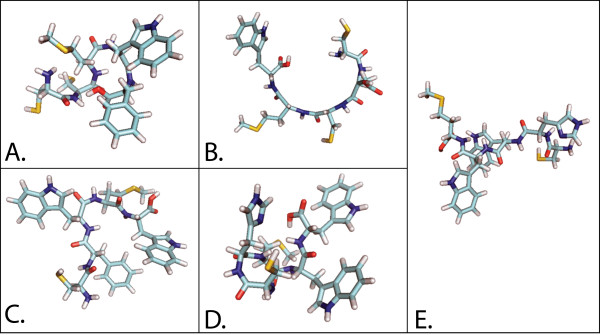
**Generated peptides**. Peptides generated by DockoMatic from single letter amino acid strings. The images show the following pentapeptides: A) CCMWF, B) CDCMW, C) CFWMW, D) CHMWW, and E) CHWWM.

**Figure 3:**
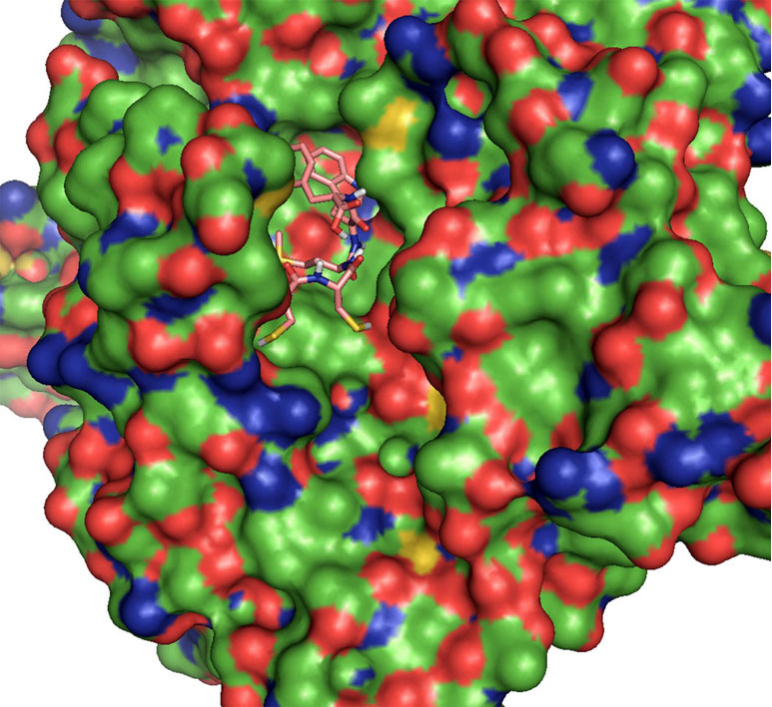
**DockoMatic output**. DockoMatic result output .pdb file image showing the best ranked (lowest binding energy) binding conformation for CCMWF in complex with Ac-AChBP as calculated by AutoDock.

For this test, the evaluation of DockoMatic made use of a 61 node Beowulf cluster at Boise State University. The files used for the testing process included: 1) the receptor .pdb file for the crystal structure of the *Aplysia californica *acetylcholine binding protein (*Ac*-AChBP) obtained from the Research Collaboratory for Structural Bioinformatics (RCSB) database at http://www.pdb.org, 2UZ6, and 2) five peptide ligands each comprised of five amino acids: CCMWF, CDCMW, CFWMW, CHMWW, and CHWWM. All five ligands were submitted as a simple text file. DockoMatic successfully created the corresponding .pdb files, viewable in Figure 2. These five .pdb files were then automatically directed and paired with the *Ac*-AChBP .pdb file and the matching .gpf file into submission ready AutoDock jobs by DockoMatic. Upon job completion, DockoMatic parsed the .dlg files into individual, ranked result .pdb files. These files were easily viewable by clicking on the PyMOL button. Figure 3 is an example of one of the created ligands docked with the *Ac*-AChBP receptor as viewed by PyMOL. In addition to individually ranked .pdb files, DockoMatic also provides a master list of ranked results for each job. This list appears as a simple text file consisting of the detailed results for each rank, such as the estimated binding energy and inhibition constant as calculated by AutoDock.

**Figure 4:**
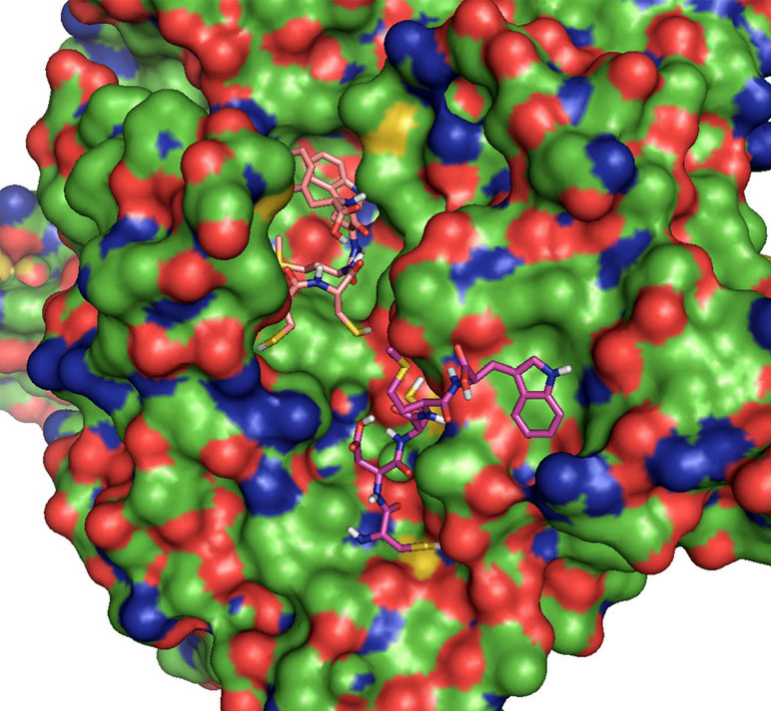
**Competitive binding**. Result for competitive binding simulation beginning with the docking of CCMWF to Ac-AChBP receptor. The best result of CCMWF with Ac-AChBP forms a new receptor-ligand complex, which is then allowed to bind to the secondary ligand, CDCMW. The lowest binding energy complex is displayed.

Two of the created peptide ligands were selected to test in a sample competitive binding experiment. One ligand was selected in the ligand box, and the other was placed in the secondary ligand field. DockoMatic then prepared the necessary files for the competitive binding experiment (see Figure 4).

### Ligand creation

Our motivation for adding the ability to automatically create peptide ligands comes from our experience using Autodock to study peptides, in particular those derived from the venom of snails of the genus Conus, which have demonstrated great potential as potent and selective ligands for myriad biological receptors [15,16]. Understanding how these peptides function has proven challenging by traditional molecular biology bench laboratory techniques, and computational modeling has evolved as a useful tool to study the interaction between peptide ligands and large biological receptors [17].

While tools exist to create peptide-based ligands, we are not aware of any that are automated. Most applications in this area require users to manually create the peptide ligand by placing and rotating individual amino acids using a mouse. This involves such tasks as selecting each amino acid, from a group of the 20 common amino acids, in order to create a peptide with the correct sequence. Second, the oxidation state of each peptide is set to be $NH_{3}^{+}$ for the amine terminus, and *COO^- ^*for the carboxy terminus. The user then selects parameters for the program to create a three-dimensional coordinate structure for each peptide, saving them in .pdb file format. Traditionally a molecular modeling program, such as Spartan [18], is used to do this.

An experienced user takes about 2.5 minutes to create a pentapeptide using Spartan. It required approximately 12 minutes to create all five ligands for the test conducted. A user unfamiliar with molecular modeling software could take significantly longer to create these ligands. This time is dependent upon the length of the amino acid sequence, adding more amino acids generally causes the creation time to grow linearly with the number of amino acids.

In contrast, DockoMatic can prepare the same five pentapeptides in approximately 34 seconds of computational time. For DockoMatic, essentially no user time is required for ligand creation. All that DockoMatic requires is a single string representation of the amino acid sequence.

### Concurrent job submission and time

Our testing showed that it took on the order of 31 minutes to perform all tasks required to submit the five AutoDock jobs. This includes 12 minutes for creating .pdb files for five pentapeptide ligands and 19 minutes to prepare the ligand, receptor and .gpf grid files. In contrast, the time required to perform the same sequence of events using DockoMatic consists of the time to enter the locations of the input files and press two buttons for creating and starting the jobs.

In this instance, with 5 amino acid strings listed in a ligand input file, 1 box coordinate file, and 1 receptor, it took DockoMatic approximately 16 seconds of user time to begin the five AutoDock jobs. Adding one minute to that time for grid box file creation yields a total time of 1 minute and 16 seconds.

Using AutoDock, a user is initially forced to wait while the atom affinity map files are created. This process took almost 19 minutes for the files used during testing. On the other hand, with DockoMatic, the user is unaware of this step as it happens automatically.

Once the ligands, receptor, .gpf grid files, and the affinity map files are created and prepared, the time required to run a given AutoDock job is hardware dependent. So, comparative job runtimes are not particularly meaningful in the sense that they only show differences in hardware. More relevant than the time to run a few AutoDock jobs is the user time required to manage and submit the AutoDock jobs.

Assuming that the ligand list and the template box coordinate file have been generated, creating and running large numbers of jobs with DockoMatic takes essentially the same amount of user time as 1 job. The process involves browsing for the correct ligand, receptor, and grid box files followed by job submission. For instance, if using a list of 256 ligands, the only difference to the experiment above would be the name of the ligand list file.

Based on the previous experiment, attempting the same task of starting 256 docking jobs manually would require approximately 26 hours of user time before the jobs could be submitted to AutoDock. Ten of those hours would be dedicated to ligand creation alone.

## Conclusions

We have demonstrated that DockoMatic: 1) provides an intuitive GUI for the user, 2) automates AutoDock job setup, submission, and management for high throughput docking experiments, 3) can automate experiments involving multiple ligands for competitive binding, 4) tracks multiple jobs in realtime on a cluster, 5) automates the creation of pdb files for pentapeptide ligands, 6) enables easy viewing of ranked results in individual .pdb files, and 7) helps with analysis of results. DockoMatic eliminates many of the mundane tasks involved in using AutoDock to perform simulated docking experiments, providing a useful tool for any laboratory interesting in molecular docking, especially those interested in peptide ligands. In our labs, DockoMatic has proven useful for all levels of users, from experienced to novice. All that is required from the user is the list of ligands, a receptor file, and a template grid box coordinate file. Once these have been submitted to DockoMatic, a push of a button is all that's necessary to create peptide ligands, load required AutoDock files, select output directories, and to begin processing. This frees the user from the menial task of job setup, creation, and management, allowing them to perform additional lab duties.

## Availability and requirements

• Project Name: DockoMatic

• Project home page: https://sourceforge.net/projects/dockomatic

• Operating System: Linux

• Programming Languages: Java, Perl

• License: LGPL

## Competing interests

The authors declare that they have no competing interests.

## Authors' contributions

CB created all Perl scripts, as well as all the Java code for the GUI. AutoDock was not modified, nor were any of the Python scripts from MGLTools. RJ created the individual amino acid files used for ligand creation. RJ also provided all the details necessary about how to combine the amino acids into ligands and run the other Python scripts to prepare the files for use with AutoDock. Timing and accuracy testing of DockoMatic was performed by RJ. TA and OM came up with the original idea, and TA, OM, and GH provided advice and direction regarding details of the project. RJ, TA, GH, and OM provided editorial contributions to the manuscript. All authors have read and approved the final manuscript.

## References

[B1] Kitchen D Decornez H Furr J Bajorath J Docking and scoring in virtual screening for drug discovery: methods and applications Nature 2004 3 935 949 10.1038/nrd1549 15520816

[B2] Kontoyianni M McClellan L Sokol G Evaluation of docking performance: comparative data on docking algorithms J Med Chem 2004 47 558 565 10.1021/jm0302997 14736237

[B3] Morris G Goodsell D Halliday R Huey R Hart W Automated docking using a Lamarckian genetic algorithm and an empirical free energy function J Comput Chem 1998 19 1639 1662 10.1002/(SICI)1096-987X(19981115)19:14<1639::AID-JCC10>3.0.CO;2-B

[B4] Chemical Computing Group MOE http://www.chemcomp.com/

[B5] Gold Version 1.2 http://www.ccdc.cam.ac.uk/products/life_sciences/gold/

[B6] Ewing T Makino S Skillman A Kuntz I DOCK 4.0: search strategies for automated molecular docking of flexible molecule databases J Comput Aided Mol Des 2001 15 411 428 10.1023/A:1011115820450 11394736

[B7] Friesner R Banks J Murphy R Halgren T Klicic J Mainz D Repasky M Knoll E Shelley M Perry J Shaw D Francis P Shenkin P Glide: A new approach for Rapid, Accurate Docking and Scoring. 1. Method and assessment of docking accuracy J Med Chem 2004 47 1739 1749 10.1021/jm0306430 15027865

[B8] Cavasotto C Clemente-Gallardo J, Moreno Y, lorenzo JS, Velasquez-Campoy A Ligand docking and virtual screening in structure-based drug discovery From Physics to Biology 2006 34 49

[B9] Zhang S Kumar K Jiang X Wallqvist A Reifman J DOVIS: an implementation for high-throughput virtual screening using AutoDock BMC Bioinformatics 2008 9 126 10.1186/1471-2105-9-126 18304355 PMC2267697

[B10] PyMOL http://www.pymol.org

[B11] AutoDock Tools http://autodock.scripps.edu/resources/adt

[B12] Li C Xu L Wolan DW Wilson LA Olson AJ Virtual Screening of Human 5-Aminoimidazole-4-carboxamide Ribonucleotide Transformylase against the NCI Diversity Set by Use of AutoDock to Identify Novel Nonfolate Inhibitors J Med Chem 2004 47 6881 6690 10.1021/jm049504o 15615517

[B13] OpenBabel http://openbabel.sourceforge.net/

[B14] Swarm http://biowulf.nih.gov/apps/swarm.html

[B15] Jacob R McDougal O The M-superfamily of conotoxins: a review Cellular and molecular life sciences 2010 1 17 10.1007/s00018-009-0125-0 PMC3741454 19705062

[B16] Millard E Daly N Craik D MINIREVIEW: Structure-activity relationships of alpha-conotoxins targeting neuronal nicotinic acetylcholine receptors European Journal of Biochemistry 2004 271 12 2320 2326 10.1111/j.1432-1033.2004.04148.x 15182347

[B17] Zoete V Grosdidier A Michielin O Docking, virtual high throughput screening and in silico fragment-based drug design Journal of Cellular and Molecular Medicine 2009 13 2 238 248 10.1111/j.1582-4934.2008.00665.x 19183238 PMC3823351

[B18] SPARTAN http://www.wavefun.com/products/spartan.html

